# Prognostic accuracy of the Stroke Rehabilitation Assessment of Movement (STREAM) scores on admission for walking independence in stroke patients at discharge and one-month follow-up

**DOI:** 10.1371/journal.pone.0319682

**Published:** 2025-03-07

**Authors:** Thamonwan Kirdthongkham, Maria Justine, Akkradate Siriphorn

**Affiliations:** 1 Department of Physical Therapy, Faculty of Allied Health Sciences, Chulalongkorn University, Bangkok, Thailand,; 2 Department of Physical Therapy, Queen Savang Vadhana Memorial Hospital, Chonburi, Thailand,; 3 Centre for Physiotherapy Studies, Faculty of Health Sciences, Universiti Teknologi MARA, Puncak Alam Campus, Selangor, Malaysia; Imperial College London, UNITED KINGDOM OF GREAT BRITAIN AND NORTHERN IRELAND

## Abstract

Gait prediction is critical in optimizing rehabilitation strategies for stroke survivors. This study evaluates the prognostic utility of the Stroke Rehabilitation Assessment of Movement (STREAM) scores, recorded at admission, for predicting walking ability at discharge and one-month follow-up. We assessed 47 stroke patients using STREAM at admission; walking independence was defined using two criteria: a Functional Ambulation Category (FAC) score >  3 and a 10-Meter Walk Test (10-MWT) speed ≥  0.4 m/s. The predictive validity of STREAM scores was analyzed using the area under the receiver operating characteristic curve (AUC). Sensitivity, specificity, and cut-off values were computed. The analysis revealed that a STREAM score above 38 at admission significantly predicted independent gait by discharge, evidenced by a high AUC of 0.897. At the one-month follow-up, a cut-off score of 29 continued to predict walking independence, with an AUC of 0.987. The subscores further enhanced predictive accuracy and highlighted the effectiveness of the STREAM assessment as a robust predictor of independent walking in stroke patients. These findings suggest the practicality of using STREAM scores to predict walking independence, which can guide the planning of more effective rehabilitation interventions. **Trial registration** TCTR20240323004 at www.thaiclinicaltrials.org.

## Introduction

Stroke remains a major global health challenge, ranking as the second leading cause of death and the third leading cause of disability worldwide [1]. Lower limb impairments and walking limitations, which affect a considerable portion of stroke survivors, significantly impact their balance and mobility. Focused rehabilitation that emphasizes motor control and early assessment of walking ability is crucial [2]. Studies show that 44.1% of stroke patients experience lower extremity motor deficits, and 57.9% have walking limitations [3]. Despite these challenges, more than half of the patients (50.9%) regain sufficient mobility to be discharged home [3]. The early phase post-stroke is critical for cerebral repair and recovery, highlighting the importance of timely and appropriate rehabilitation interventions. Such interventions can improve patient satisfaction, reduce long-term care costs, and ultimately, enhance the quality of life [4].

Research has shown that factors such as age, initial motor and functional impairments are significant predictors of walking recovery during rehabilitation [5]. For example, a study using the Berg Balance Scale indicate that patients scoring 27 or higher one month post-stroke are likely to achieve independent gait within three months [6]. Identifying key predictors, such as balance, motor control, and demographic variables, may enable clinicians to tailor interventions effectively. This knowledge is crucial for clinicians to set realistic goals, optimize therapy, and support effective discharge planning, thereby enhancing walking outcomes for stroke patients.

Evidence-based treatments that facilitate early hospital discharge are vital in acute rehabilitation settings. Physicians often rely on objective outcome measures for diagnosis, evaluation, and therapy planning. Consistency in evaluations is key to allowing physical therapists to communicate effectively and compare the efficacy of rehabilitation treatments [7]. Among these tools, the Stroke Rehabilitation Assessment of Movement (STREAM) stands out as a widely used assessment method designed to evaluate limb movement and mobility following a stroke [8]. STREAM is employed in both research and clinical practice and has proven effective across a wide spectrum of stroke severity, from mild to severe [8]. It demonstrates high inter-rater and intra-rater reliability and aligns closely with the Fugl-Meyer Assessment in terms of concurrent validity [9].

STREAM is practical, requiring no special equipment, and consists of 30 items, with 10 each dedicated to the upper extremity, lower extremity, and basic mobility. Both STREAM and the Functional Independence Measure (FIM) demonstrate moderate to high correlations with projected and actual lengths of stay in rehabilitation. Notably, STREAM shows a stronger correlation with admission length of stay compared to FIM, which could be due to STREAM’s dual focus on both impairment and function, whereas FIM emphasizes only the functional aspect [10].

Walking ability post-stroke significantly influences a patient’s quality of life. In the early stages, monitoring voluntary movement is essential for patients who are unable to perform high-functioning tasks. STREAM includes assessments of basic voluntary movements and bed mobility tasks, such as bridging, rolling to the side, standing, and taking a few sideways steps—all of which are linked to daily living activities and walking [11]. Thus, STREAM subscale items provide valuable information on both impairments and functional abilities, making it a robust tool for tracking post-stroke recovery.

Given the significant impact of stroke on mobility and the importance of early rehabilitation in promoting recovery, there is a clear need for reliable, evidence-based tools that can predict patient outcomes and guide therapeutic interventions. The STREAM assessment, with its dual focus on both impairment and function, offers a comprehensive evaluation of motor recovery and walking ability. However, its full potential as a predictive tool for walking outcomes at discharge has not been thoroughly explored. Understanding STREAM’s predictive capabilities could provide clinicians with critical insights, allowing for more targeted rehabilitation strategies and improving the precision of discharge planning. By identifying which patients are most likely to benefit from specific interventions, clinicians can optimize therapy, enhance patient outcomes, and reduce the risk of long-term disability. This study aims to fill a critical gap in the literature by evaluating STREAM’s ability to predict walking outcomes both at discharge and at a one-month follow-up. By assessing outcomes at these two critical time points, the study offers a comprehensive evaluation of STREAM’s predictive utility, providing clinicians with a robust foundation for decision-making in stroke rehabilitation planning and goal setting.

## Methods

### Study design

This prospective cohort study was conducted at Queen Savang Vadhana Memorial Hospital, Thailand, between March and May 2024, involving first-time stroke patients. During their initial visit to the physical therapy department, participants underwent the STREAM to predict walking capabilities at discharge. Subsequent assessments were conducted using the Functional Ambulation Category (FAC) and the 10-Meter Walk Test (10-MWT) at discharge and again at a one-month follow-up. Each patient received a comprehensive rehabilitation program that included physical, occupational, and speech therapy. This study was approved by the Queen Savang Vadhana Memorial Hospital’s Institutional Review Board (COE No. 041/2566) and registered with www.thaiclinicaltrials.org (registration number TCTR20240323004). The participants signed the written informed consent prior to their involvement in this study.

### Sample size

The sample size was calculated using MedCalc software version 22.007 (© 1993-2020 MedCalc Software). Parameters were set to ensure statistical significance (P <  0.05), with a statistical power of 0.8 and the ROC value incorporated as the null hypothesis. Based on prior research, it was estimated that 79% of patients would achieve independent walking [12]. Assuming an area under the curve of receiver operational curve (AUC) of 0.8 [13], 36 participants were required, with an expected dropout rate of 30%. In total, 47 participants were included in the study.

### Participants

A total of 47 first-time stroke patients were recruited for the study. The inclusion criteria were as follows: participants were aged between 20 and 80 years, including both male and female individuals. They were required to have a confirmed first-time stroke diagnosis, verified through computerized tomography (CT) or magnetic resonance imaging (MRI). Participants needed to demonstrate the ability to follow one or two simple commands, indicating sufficient cognitive function to participate in the study. Stable vital signs were assessed through clinical evaluation to ensure participants were medically stable and capable of participating in rehabilitation activities. The exclusion criteria were designed to eliminate factors that could affect the study’s outcomes related to gait or balance. These criteria included any orthopedic conditions such as severe osteoarthritis or lower extremity joint deformities that could impair mobility independently of stroke effects. Neurological conditions affecting motor function, including recurrent stroke or disorders like Parkinson’s disease and multiple sclerosis, were excluded to maintain the focus on stroke-specific outcomes. Participants were also excluded if they had recent lower limb surgical interventions (e.g., knee or hip arthroplasty) or severe vestibular disorders, which could compromise balance.

### Outcome measurements

#### Stroke Rehabilitation Assessment of Movement (STREAM).

The STREAM is a comprehensive instrument specifically designed to assess both upper and lower limb functions in post-stroke patients. Comprising 30 items, the assessment takes approximately 15 minutes to complete. The categorization of items is methodically based on the patient’s position during the evaluation: items 1-6 with the patient in a supine position, 7-21 seated, 22 standing without support, 23-25 standing with support, and 26-30 involve walking tasks [14]. The STREAM was conducted in a serene and controlled setting, with therapists giving standardized instructions to ensure consistency.

The STREAM evaluates three primary domains: The Upper and Lower Limb Voluntary Movements, each assessed on a 3-point ordinal scale (0–2), and Basic Mobility, evaluated on a 4-point ordinal scale (0–3). Both limb domains have a potential maximum score of 20 points (cumulatively 40), while the mobility domain can achieve up to 30 points. When items were unsuitable for evaluation due to restrictions such as pain or range of motion, they were marked “X” and excluded from the final scoring. Each domain score was converted to percentages, and the overall STREAM score was derived from the average of these percentage scores [14].

#### Functional Ambulatory Category (FAC).

The FAC is a reliable ordinal scale [15, 16], excellent test-retest and interrater reliability also good concurrent and predictive validity in patient after stroke [17]. It is used to classify the level of physical assistance a patient requires for safe ambulation after a stroke. The FAC assessment was integrated into the 10-Meter Walk Test (10-MWT), where participants’ walking abilities were evaluated over a 10-meter distance. Following the 10-MWT, participants were further assessed on their ability to ascend and descend stairs, providing additional insights into functional mobility on uneven and challenging surfaces. This stair assessment aimed to evaluate participants’ physical coordination, strength, balance, and safety during more complex tasks that simulate real-life scenarios. Breaks were allowed to ensure participant comfort. The FAC uses a six-tiered categorization: 0, requires continuous support from one or two persons; 1, requires continuous or intermittent support from one person; 2, needs supervision or verbal cues, with or without physical contact; 3, can ambulate independently on level surfaces; 4, can walk independently on most surfaces but may need assistance on stairs or uneven surfaces; 5, completely independent, even on challenging terrains. In this assessment, a FAC score above 3 indicates “Independent” ambulation, while a score of 3 or below indicates “Dependent” ambulation. For participants scoring at levels 4 and 5, their ability to ascend and descend stairs was further evaluated.

#### 10-Meter Walk Test (10-MWT).

The 10-MWT is a reliable assessment tool to evaluate walking ability in stroke patients. Markers were placed, setting out a 14-meter path with specific points at the start, 10 meters, and the end. Participants began their walk 2 meters before the starting line, which allowed them to establish a steady walking rhythm by the time they reach the timed section. Participants were instructed to walk comfortably, while a researcher, accompanied them, without interfering. Two trials were conducted, and speed was calculated using the 10-meter distance. A speed below 0.4 m/s was classified as a “dependent ambulator”, while any speed exceeding this threshold categorized them as an “independent ambulator” [18].

### Research protocol

During the initial visit to the physical therapy department, participants underwent the STREAM to predict their walking capabilities at discharge. Subsequent assessments were conducted using the FAC and the 10-MWT at discharge and a one-month follow-up.

Data collected included both demographic and clinical details, such as age, gender, type of stroke, time from stroke onset to assessment, the affected body side, the National Institutes of Health Stroke Scale (NIHSS) [19], and the Modified Rankin Scale (mRS) [20].

Upon discharge from inpatient rehabilitation, participants were evaluated using both the FAC and the 10-MWT. Based on these assessments, participants were classified into two groups: 1) Independent ambulators: defined by an FAC score greater than 3 and a 10-MWT speed of 0.4 m/s or faster; 2) Dependent ambulators: defined by an FAC score of 3 or less and a 10-MWT speed below 0.4 m/s.

The same designated physical therapist conducted these assessments to ensure consistency. A follow-up evaluation was performed one month after discharge to examine progress in rehabilitation outcomes, utilizing the FAC and the 10-MWT to quantify changes in walking ability and functional mobility.

### Statistical analysis

Receiver Operating Characteristic (ROC) curves were used to evaluate the sensitivity, specificity, and accuracy of the STREAM score at admission in predicting independent walking at discharge and at the one-month follow-up, utilizing MedCalc software (MedCalc Software Ltd, Belgium). The area under the curve (AUC) was used to quantify the accuracy of the STREAM score as a predictive tool. An AUC value close to 1 indicated a very good predictive accuracy. AUC values above 0.90 were considered to reflect high accuracy, values between 0.70 and 0.90 moderate accuracy, and values between 0.50 and 0.69 low accuracy. An AUC below 0.50 suggested the model’s predictions were no better than random chance.

We compared the areas under the ROC curves (AUCs) for the STREAM total and subscale scores using MedCalc software. The comparison of AUCs was performed using the DeLong test, a nonparametric method for comparing the areas under two or more correlated ROC curves, as described by DeLong et al. (1988) [21]. This approach evaluates the statistical significance of the observed differences in AUC values. P-values <  0.05 were considered statistically significant.

The Youden Index, calculated as sensitivity +  specificity – 1, was used to determine the cut-off value that maximized both sensitivity and specificity. The likelihood ratio was also used to evaluate the predictive power of the STREAM score. Descriptive statistics, including means, standard deviations (SDs), and proportions, were calculated.

## Results

The study included 47 participants with a mean age of 53.28 years (SD =  10.10). The majority of participants had ischemic strokes (78.70%), with the rest experiencing hemorrhagic strokes (21.30%). All participants were of Asian ethnicity, reflecting the demographics of the study region. The mean NIHSS score was 5.96 (SD =  4.47), and the average length of hospital stay was 5.28 days (SD =  2.58). Although the interval between admission and discharge was relatively short, the predictive validity of STREAM scores was established by assessing walking independence at both discharge and the one-month follow-up. This dual-timepoint evaluation ensures that the STREAM scores provide robust predictions of both short-term and medium-term walking outcomes. The mean total STREAM score was 43.52 (SD =  23.78), with upper extremity (UE), lower extremity (LE), and mobility domain scores detailed in Table 1.

**Table 1:** Demographics characteristic of all participants.

Variable	Total (N = 47)
Age, y, mean (SD)	53.28 (10.10)
Sex, n (%)	
Male	25 (53.20)
Female	22 (46.80)
Type of stroke n (%)	
Ischemic	37 (78.70)
Hemorrhagic	10 (21.30)
NIHSS, mean (SD)	5.96 (4.47)
mRS, n (%)	
0	5 (10.60)
1	15 (31.90)
2	14 (29.80)
3	5 (10.60)
4	8 (17.00)
Baseline STREAM, mean (SD)	
STREAM (Total)	43.52 (23.78)
STREAM (UE)	13.00 (7.36)
STREAM (LE)	13.81 (7.15)
STREAM (Mobility)	18.06 (10.42)

NIHSS: National Institutes of Health Stroke Scale; mRS: Modified Rankin Scale; STREAM: Stroke Rehabilitation Assessment of Movement; LE: Lower Extremity; UE: Upper Extremity.

At discharge, the independent ambulation group (n =  21) had a significantly higher mean STREAM total score of 62.95 (SD =  7.77) compared to the dependent ambulation group (n =  26), which had a mean score of 31.08 (SD =  22.81). This difference was statistically significant (P <  0.0001). At the one-month follow-up, the independent group (n =  29) maintained a higher mean STREAM score of 61.04 (SD =  8.69), while the dependent group (n =  18) had a mean score of 16.79 (SD =  13.26), showing a significant difference (P <  0.0001) (see Table 2 for details).

**Table 2:** Mean of Stroke Rehabilitation Assessment of Movement (STREAM) score between independent and dependent groups.

Variable	Independent ambulators		Dependent ambulators			
	n	Mean (SD)	n	Mean (SD)	Difference (95% CI)	P ^a^
At discharge						
STREAM (Total)	21	62.95 (7.77)	26	31.08 (22.81)	–31.88 (–42.38 to –21.37)	**<0.001** *
STREAM (UE)	21	17.43 (3.14)	26	10.23 (8.22)	–7.20 (–11.03 to –3.37)	**<0.001** *
STREAM (LE)	21	19.00 (2.24)	26	9.62 (7.00)	–9.38 (–12.59 to –6.18)	**<0.001** *
STREAM (Mobility)	21	26.52 (3.89)	26	11.23 (8.89)	–15.29 (–19.50 to –11.09)	**<0.001** *
At one-month follow-up						
STREAM (Total)	29	61.04 (8.69)	18	16.79 (13.26)	–44.26 (–51.46 to –37.05)	**<0.001** *
STREAM (UE)	29	17.75 (3.06)	18	5.71 (6.16)	–12.04 (–15.06 to –9.01)	**<0.001** *
STREAM (LE)	29	18.25 (2.91)	18	5.29 (4.73)	–12.96 (–15.47 to –10.46)	**<0.001** *
STREAM (Mobility)	29	25.04 (5.24)	18	5.79 (3.98)	–19.26 (–22.54 to –15.97)	**<0.001** *

^a^T-test; STREAM = Stroke Rehabilitation Assessment of Movement; UE = Upper Extremity; LE = Lower Extremity

**Fig 1:**
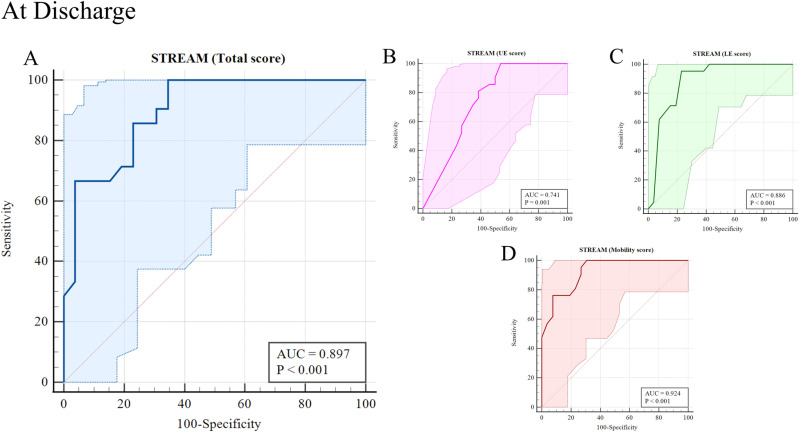
Receiver operating characteristic (ROC) curve analyses of assessed independent ambulation of stroke patients with Stroke Rehabilitation Assessment of Movement (STREAM) and subscale score at discharge.

**Fig 2:**
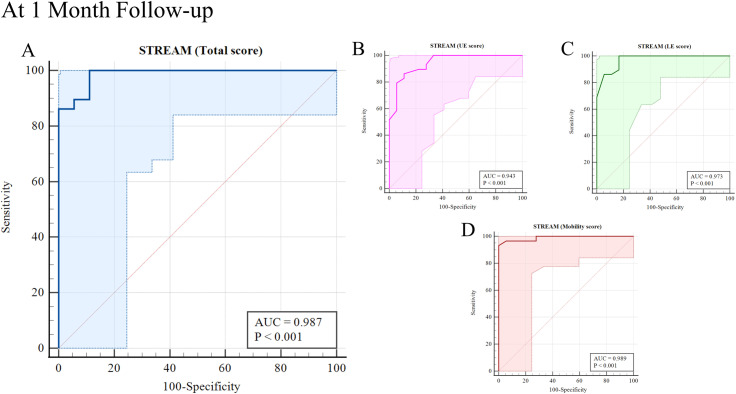
Receiver operating characteristic (ROC) curve analyses of assessed independent ambulation of stroke patients with Stroke Rehabilitation Assessment of Movement (STREAM) and subscale score at one-month follow-up.

The ROC curve analysis demonstrated high diagnostic accuracy for predicting independent ambulation. At discharge, the AUC for the STREAM total score was 0.90. Among the subdomains, the AUC values were 0.74 for UL, 0.89 for LE, and 0.92 for mobility, with the mobility domain demonstrating the highest predictive accuracy. At the one-month follow-up, the AUC for the STREAM total, UE, LE, and mobility scores was 0.99, 0.94, 0.98, and 0.99, respectively. These results indicate that the STREAM and its subscales are reliable predictors of independent ambulation in stroke patients (Figs 1 and 2 and Table 3).

**Table 3:** Receiver operating characteristic curve (ROC) analysis for predicting independent ambulation in stroke patients at discharge and one-month follow-up.

	AUC (95%CI)	z statistic	*P*-value
**At discharge**			
STREAM (Total)	0.90 (0.77 to 0.96)	9.06	**<0.001** *
STREAM (UE)	0.74 (0.59 to 0.86)	3.37	**<0.001** *
STREAM (LE)	0.89 (0.76 to 0.96)	7.87	**<0.001** *
STREAM (Mobility)	0.92 (0.81 to 0.98)	11.89	**<0.001** *
**At one-month follow-up**			
STREAM (Total)	0.99 (0.90 to 1.00)	42.74	**<0.001** *
STREAM (UE)	0.94 (0.83 to 0.99)	13.92	**<0.001** *
STREAM (LE)	0.98 (0.88 to 0.99)	25.77	**<0.001** *
STREAM (Mobility)	0.99 (0.90 to 1.00)	46.71	**<0.001** *

The cut-off values, sensitivity, specificity, and likelihood ratios for predicting independent ambulation, based on the STREAM total and subscale scores, are presented in Table 4. These values highlight that the mobility domain consistently performed well, with high sensitivity and specificity at both discharge and follow-up.

**Table 4:** Cut-off values, sensitivity, and specificity of ambulation prediction by Stroke Rehabilitation Assessment of Movement (SRTEAM) and subscale scores.

	Cut-off	Youden index	Sensitivity (95%CI)	Specificity (95%CI)	LR + (95%CI)	LR–(95%CI)
**At discharge**						
STREAM (Total)	>38	0.65	100.00 (83.90–100.00)	65.38 (44.30–82.80)	2.89 (1.70–4.90)	0.00 (0.00–0.00)
STREAM (UE)	>10	0.46	100.00 (83.90–100.00)	46.15 (26.60–66.60)	1.86 (1.30–2.65)	0.00 (0.00–0.00)
STREAM (LE)	>16	0.72	95.24 (76.20–99.90)	76.92 (56.40–91.00)	4.13 (2.03–8.38)	0.06 (0.01–0.42)
STREAM (Mobility)	>17	0.69	100.00 (83.90–100.00)	69.23 (48.20–85.70)	3.25 (1.83–5.78)	0.00 (0.00–0.00)
**At one-month follow-up**						
STREAM (Total)	>29	0.89	100.00 (88.10–100.00)	88.89 (65.30–98.60)	9.00 (2.44–33.24)	0.00 (0.00–0.00)
STREAM (UE)	>14	0.75	86.21 (68.30–96.10)	88.89 (65.30–98.60)	7.76 (2.08–28.89)	0.16 (0.06 – 0.39)
STREAM (LE)	>10	0.83	100.00 (88.10–100.00)	83.33 (58.60–96.40)	6.00 (2.14–16.86)	0.00 (0.00 – 0.00)
STREAM (Mobility)	>18	0.93	93.10 (77.20– 99.20)	100.00 (81.50–100.00)	Approaches infinity	0.069 (0.02 – 0.26)

Comparison of AUC values in Table 5 shows that at discharge, the STREAM upper extremity (UE) score had significantly lower diagnostic accuracy compared to the total score, lower extremity (LE) score, and mobility score. However, there was no significant difference in accuracy between the total, LE, and mobility subscales. At the one-month follow-up, no significant differences were observed between the total STREAM score and its subscales.

**Table 5:** Comparing the predictability of STREAM sub-scale scores at discharge and one-month follow-up. The AUC values were compared using the DeLong test, a nonparametric method for comparing ROC curves, as described by DeLong et al. (1988).

	Difference between AUC	95% CI	z statistic	P-value
At discharge				
STREAM (Total) vs. STREAM (UE)	0.16	0.07 to 0.24	3.46	**<0.001** *
STREAM (Total) vs. STREAM (LE)	0.01	–0.05 to 0.07	0.38	0.706
STREAM (Total) vs. STREAM (Mobility)	0.03	–0.01 to 0.06	1.41	0.157
STREAM (UE) vs. STREAM (LE)	0.14	0.04 to 0.25	2.63	**0.009** *
STREAM (UE) vs STREAM (Mobility)	0.18	0.08 to 0.29	3.38	**<0.001** *
STREAM (LE) vs. STREAM (Mobility)	0.04	–0.03 to 0.11	1.03	0.303
At one-month follow-up				
STREAM (Total) vs. STREAM (UE)	0.04	–0.00 to 0.09	1.83	0.068
STREAM (Total) vs. STREAM (LE)	0.013	–0.01 to 0.04	1.07	0.283
STREAM (Total) vs. STREAM (Mobility)	0.003	–0.02 to 0.02	0.26	0.793
STREAM (UE) vs. STREAM (LE)	0.030	–0.02 to 0.08	1.07	0.286
STREAM (UE) vs STREAM (Mobility)	0.046	–0.01 to 0.11	1.47	0.141
STREAM (LE) vs. STREAM (Mobility)	0.02	–0.01 to 0.05	1.01	0.313

## Discussion

This research aimed to explore the predictive capability of the STREAM for assessing walking outcomes at the time of discharge from inpatient rehabilitation and one-month post-stroke. The findings confirm that STREAM, along with its subscale scores, robustly predicts independent walking ability. The results highlight the effectiveness of the STREAM as a two-dimensional performance measure that evaluates both impairment and functional levels, accurately predicting walking ability both at discharge and during the one-month follow-up.

The typical gait cycle consists of two main phases—swing and stance—further divided into eight sub-phases, including heel strike, foot flat, and heel off [22]. STREAM evaluates critical movements necessary for walking, such as hip flexion during sitting, knee extension with ankle dorsiflexion, and knee flexion with hip extension. By assessing these impairments, STREAM and its subscale scores are highly effective in predicting walking proficiency.

ROC curve analysis revealed that STREAM total scores above 38, UE scores above 10, LE scores above 16, and Mobility scores above 17 at admission are strong predictors of independent gait at discharge and one-month post-stroke. These findings highlight the importance of early motor control and balance assessments in forecasting recovery [23]. This study also provides more precise estimates of the likelihood and cut-off points based on initial impairment. For instance, patients with STREAM Mobility scores greater than 17 at admission have a high probability of achieving independent ambulation and may benefit from intensive rehabilitation.

The STREAM mobility subscale, which assesses basic ADL movements such as rolling, sitting up, standing, and walking, aligns with Ouellette et al., who found that motor FIM and other ADL-related scores at admission strongly predict community discharge [24]. Our study also demonstrated the strong predictive value of the STREAM LE score at both discharge and one-month follow-up. At discharge, a score greater than 16 had 95.24% sensitivity, 76.92% specificity, an LR + of 4.13, and an LR- of 0.06. At one-month, a score above 10 provided 100% sensitivity, 83.33% specificity, an LR + of 6, and an LR- of 0. These results align with previous research, such as the Motricity Index leg score, where a score above 25 predicted a 98% chance of regaining independence within six months [12].

A previous study has also demonstrated significant correlations between upper and lower limb motor impairments, trunk control, FIM, motor FIM, and walking recovery as measured by FAC [5]. Walking effectiveness depends on factors such as balance, coordination, and lower extremity control. Knee extension on the paretic side, for example, has been identified as a key predictor of step-through gait patterns in sub-acute stroke patients [25]. Achieving walking ability is a key rehabilitation goal, and a higher STREAM score at admission predicts better walking outcomes at both discharge and the one-month follow-up.

Interestingly, upper extremity function did not significantly affect balance or gait directly. However, the STREAM UE subscale scores were still moderately to highly predictive of walking ability at discharge and follow-up, with AUCs of 0.74 (95% CI 0.59–0.86, P <  0.001) at discharge and 0.94 (95% CI 0.83–0.99, P <  0.001) at follow-up. Mechanical analysis suggests that arm swings during walking stabilize body rotation, aiding in balance and reducing energy expenditure. The rhythmic movement of the arms enhances muscle coordination in the lower limbs, improving gait dynamics and efficiency [26]. Upper extremity function also correlates with activities of daily living, such as dressing, toileting, and mobility, as reflected in FIM motor scores [27]. This highlights the role of upper limb motor function in overall mobility and lower limb movement. The moderate accuracy of the STREAM UE subscale scores highlights the importance of upper limb function in maintaining postural balance and performing ADLs [28].

To assess walking ability at discharge and follow-up, the FAC and 10MWT were used to categorize patients into independent or dependent ambulation groups. While the FAC effectively evaluates walking on various surfaces and stairs, it may not fully reflect the challenges of navigating community environments. Similarly, gait speeds below and above 0.4 m/s can serve as limiting indicators. The FIM is often favored for assessing gait, as it evaluates a patient’s ability to perform daily activities comprehensively [6].

Understanding impairments and outcomes is crucial for establishing clinical pathways and optimizing rehabilitation timing. STREAM and its subscale cut-off values have proven to be effective in predicting walking ability in stroke patients, making them valuable for discharge and one-month follow-up evaluations. However, the STREAM UE subscale shows significant variability in its predictive power at discharge, and further refinement is needed. Our findings, consistent with previous studies, emphasize the importance of initial motor impairment and mobility in post-stroke ambulation independence [5,12,29,30]. Recovery begins within one week to one month post-stroke [31], reinforcing the need for early detection and tailored rehabilitation focused on walking function.

Accurate prediction of walking independence at discharge is crucial for effective stroke rehabilitation planning. While STREAM provides a dual focus on impairment and function, other clinical tools and assessments have demonstrated strong predictive utility for walking outcomes. For example, the Trunk Control Test (TCT) and the knee extension strength-to-body weight ratio on the unaffected side predicted walking ability with over 91% accuracy when combined [32]. Similarly, the Modified Rivermead Mobility Index (MRMI) score, measured on day 3 post-admission, predicted walking independence at 28 days post-admission with an AUC of 0.88 [33]. Other tools, such as the Functional Independence Measure (FIM) and Berg Balance Scale (BBS), assess functional independence and balance, respectively, with lower scores at admission strongly predicting limited walking ability [34]. Additionally, the Motor Assessment Scale (MAS) has demonstrated predictive utility for functional outcomes, particularly the MAS gait (MAS-5) and rolling (MAS-1) items, which, when combined with age and prestroke residential status, accurately predicted whether patients were discharged to home with 87.3% accuracy in a multisite study [35]. STREAM’s versatility makes it uniquely positioned to provide a holistic evaluation of impairment and function. However, future studies should directly compare STREAM’s predictive performance with these established tools to determine its relative advantages and potential integration into broader rehabilitation frameworks.

Incorporating advanced technologies and comprehensive assessments can significantly enhance stroke rehabilitation outcomes. While this study focused on the predictive value of STREAM scores for walking independence, integrating additional prognostic factors, artificial intelligence (AI) and machine learning (ML) models could provide a more holistic evaluation. For instance, Korkmaz et al. (2022) demonstrated that cardiac-electrophysiological measures offer valuable insights into stroke prognosis, complementing motor-based evaluations [36]. Additionally, a systematic review by Zu et al. (2023) evaluated ML models designed to predict rehabilitation outcomes in stroke patients. The study found that while some models achieved optimal Area Under the Curve (AUC) values between 0.63 and 0.91, all were rated as having a high or unclear risk of bias, often due to inappropriate data sources or analysis processes [37]. Recent advancements, such as the XGBoost model, have demonstrated superior performance, achieving an accuracy of 87.82% in predicting walking independence and identifying key predictors like age, Fugl-Meyer Assessment for the Lower Extremity (FMA-LE), Functional Ambulation Category (FAC), and lower limb spasticity [38]. Furthermore, Chandrabhatla et al. (2023) reviewed FDA-approved AI and ML technologies for stroke management, highlighting their potential to improve diagnostic accuracy and patient outcomes [39]. By integrating such technologies and assessments, healthcare providers may be better equipped to tailor rehabilitation strategies to individual patient needs, ultimately improving outcomes.

### Strengths and limitations

A key strength of this study is its thorough evaluation of STREAM’s ability to predict walking outcomes at discharge and one-month follow-up, utilizing ROC curve analysis to provide precise cut-off values. The results are reinforced by strong statistical measures, including sensitivity, specificity, LR + , and LR–.

However, a key limitation of this study is the relatively small sample size. The sample size of 47 participants was determined using MedCalc software, incorporating statistical parameters such as a significance level of P <  0.05, statistical power of 0.8, and an expected dropout rate of 30%. While this sample size meets the methodological requirements for this study, its relatively small scale may limit the generalizability of the findings to broader or more diverse populations. Future research with larger, multicenter cohorts is recommended to confirm these results and enhance external validity.

Another limitation of this study is its focus on acute and subacute stroke cases. While this focus allowed us to explore critical early recovery phases, it limits the applicability of our findings to chronic stroke patients. Chronic stroke populations represent a substantial portion of stroke survivors, and future research should evaluate the predictive utility of STREAM scores in these later stages of recovery to broaden the clinical relevance of our findings.

The STREAM assessment used in this study focuses exclusively on motor function, without evaluating critical non-motor factors such as cognitive and emotional variables. This exclusion limits the comprehensiveness of the tool, as cognitive and emotional states play a significant role in stroke rehabilitation and recovery outcomes. Future research should integrate assessments of these factors to provide a more holistic understanding of predictors of walking independence and recovery trajectories.

This study was conducted at a single institution, Queen Savang Vadhana Memorial Hospital. While this setting allowed for consistent data collection and controlled variables, it limits the external validity of the findings, as results may be influenced by local clinical practices and patient demographics. Additionally, the lack of diversity in the study population—where all participants were of Asian ethnicity—may limit the generalizability of the findings to other ethnic groups. Future multicenter studies involving diverse populations and clinical environments are recommended to enhance the generalizability and applicability of the findings.

Socio-economic data were not collected as part of this study, which may limit the ability to evaluate the influence of socio-economic factors on recovery outcomes. Future research should incorporate socio-economic data to provide a more nuanced understanding of predictors and their impact on walking independence.

This study utilized a single designated therapist to conduct all assessments, which ensured uniformity in scoring and minimized inter-rater variability. However, this approach may introduce bias, as the therapist’s awareness of the study objectives could influence the results. While standardized protocols and rigorous training were implemented to uphold objectivity, future research should consider employing multiple assessors or incorporating blinded evaluations to further mitigate potential bias and enhance the robustness of the findings.

### Clinical implications

The findings of this study have significant clinical implications. STREAM, with its cut-off values, provides clinicians with a practical tool for predicting walking outcomes in stroke patients, facilitating personalized rehabilitation planning. High STREAM scores at admission highlight the value of early assessment, enabling clinicians to identify patients likely to achieve independent ambulation and target intensive rehabilitation for those at risk of prolonged dependence. For patients below the cut-off, rehabilitation efforts can focus on improving walking performance. The STREAM mobility subscale’s strong predictive power suggests that early rehabilitation should prioritize mobility, trunk control, balance, and lower extremity function to enhance walking independence.

## Supporting information

S1 file Raw data. (xls)
